# Meta-analysis of miRNA expression profiles for prostate cancer recurrence following radical prostatectomy

**DOI:** 10.1371/journal.pone.0179543

**Published:** 2017-06-26

**Authors:** Elnaz Pashaei, Elham Pashaei, Maryam Ahmady, Mustafa Ozen, Nizamettin Aydin

**Affiliations:** 1 Department of Computer Engineering, Yildiz Technical University, Istanbul, Turkey; 2 Department of Computer Engineering and IT, Payame Noor University, Tehran, Iran; 3 Department of Pathology & Immunology Baylor College of Medicine, Houston, Texas, United States of America; University of South Alabama Mitchell Cancer Institute, UNITED STATES

## Abstract

**Background:**

Prostate cancer (PCa) is a leading reason of death in men and the most diagnosed malignancies in the western countries at the present time. After radical prostatectomy (RP), nearly 30% of men develop clinical recurrence with high serum prostate-specific antigen levels. An important challenge in PCa research is to identify effective predictors of tumor recurrence. The molecular alterations in microRNAs are associated with PCa initiation and progression. Several miRNA microarray studies have been conducted in recurrence PCa, but the results vary among different studies.

**Methods:**

We conducted a meta-analysis of 6 available miRNA expression datasets to identify a panel of co-deregulated miRNA genes and overlapping biological processes. The meta-analysis was performed using the ‘MetaDE’ package, based on combined P-value approaches (adaptive weight and Fisher's methods), in R version 3.3.1.

**Results:**

Meta-analysis of six miRNA datasets revealed miR-125A, miR-199A-3P, miR-28-5P, miR-301B, miR-324-5P, miR-361-5P, miR-363*, miR-449A, miR-484, miR-498, miR-579, miR-637, miR-720, miR-874 and miR-98 are commonly upregulated miRNA genes, while miR-1, miR-133A, miR-133B, miR-137, miR-221, miR-340, miR-370, miR-449B, miR-489, miR-492, miR-496, miR-541, miR-572, miR-583, miR-606, miR-624, miR-636, miR-639, miR-661, miR-760, miR-890, and miR-939 are commonly downregulated miRNA genes in recurrent PCa samples in comparison to non-recurrent PCa samples. The network-based analysis showed that some of these miRNAs have an established prognostic significance in other cancers and can be actively involved in tumor growth. Gene ontology enrichment revealed many target genes of co-deregulated miRNAs are involved in “regulation of epithelial cell proliferation” and “tissue morphogenesis”. Kyoto Encyclopedia of Genes and Genomes (KEGG) analysis indicated that these miRNAs regulate cancer pathways. The PPI hub proteins analysis identified CTNNB1 as the most highly ranked hub protein. Besides, common pathway analysis showed that TCF3, MAX, MYC, CYP26A1, and SREBF1 significantly interact with those DE miRNA genes. The identified genes have been known as tumor suppressors and biomarkers which are closely related to several cancer types, such as colorectal cancer, breast cancer, PCa, gastric, and hepatocellular carcinomas. Additionally, it was shown that the combination of DE miRNAs can assist in the more specific detection of the PCa and prediction of biochemical recurrence (BCR).

**Conclusion:**

We found that the identified miRNAs through meta-analysis are candidate predictive markers for recurrent PCa after radical prostatectomy.

## Introduction

Prostate cancer (PCa) is the most diagnosed malignancy and the second most reason of cancer-related death for the men over the age of 50 in the western countries [1]. The prostate-specific antigen (PSA) is the most reliable biomarker for PCa, which is helpful for diagnosis, screening, and follow-up after surgery. For treatment of PCa, two treatment methods, radiation therapy or radical prostatectomy (RP) and hormone ablation therapy are used. Yet, these methods do not provide enhanced survival rates and nearly 30% of patients experience a biochemical recurrence with enhanced PSA levels after curative treatment of RP [2]. Moreover, metastatic and advanced tumors of PCa respond very poorly to chemotherapy [3]. All these facts emphasize the significance of developing early diagnostic biomarkers for PCa progression. Identifying effective predictors of tumor recurrence after the surgical operation to determine whether treatment is required or not is a main challenge in the PCa research. To predict biochemical recurrence (BCR) of PCa after RP and develop effective predictors of tumor recurrence, multiple studies have been conducted for gene expression profiling [4–6]. Recently, numerous studies have been published which show that the alterations in microRNAs are associated with PCa initiation and progression [7–9].

The miR-1, miR-133b, miR-519d, and miR-647 are new biomarkers with prognostic and diagnostic value for recurrence of PCa, which have been identified through miRNA expression profiling [10, 11]. The miR-449b, miR-21, miR-141 and miR-221 are also known as putative prognostic or predictive markers in PCa recurrence after RP [12–14].

Meta-analysis utilizes statistical methods to contrast and combines results from multiple studies in the hope of increasing the statistical power and reproducibility over individual studies and identifying patterns across studies [15]. A limited number of studies [1, 10–14, 16, 17] has been conducted on microRNA expression profiles to distinguish recurrent from non-recurrent prostate tumor tissues and to identify novel biomarkers for prediction of PCa progression. The average differential expression level (fold change) and some level of significance as measured by the t-test are common procedures for identifying the biomarkers. These miRNA microarray data sets provide a rich resource for genome-wide information on PCa progression and make an ideal chance to perform a meta-analysis study. We assumed that a meta-analysis of some miRNA expression datasets of PCa progression can give a potentially significant list of co-deregulated miRNAs in PCa progression, which is important to specify pathways in which the miRNAs of interest and their target genes are involved. To increase the probability of revealing truly significant deregulated miRNA genes, which should have higher potentials to be utilized as consistent biomarkers for the disease, we analyzed miRNA expression profile in PCa progression considering 5 studies (6 datasets). This meta-analysis increases the significance of the results.

## Materials and methods

### Literature analysis

There are a limited number of reports in the literature studied miRNAs in PCa progression. We systematically queried for these studies from PubMed database.

The following Medical Subjective Heading (MeSH) and Embase tree were used: “recurrence” or “recurrence” and “prostatic neoplasms” or “prostate cancer” and “micrornas” or “microRNA” and “gene expression” or “expression”. In addition, publicly available microRNA data sets were searched by “RISmed” package in R to ensure no relevant studies were missed. Through database searching, a total of 24 studies was identified. Of these, 19 studies were retained after rejecting repetition. According to the title and abstract, a total of 14 studies was excluded. Review, case report, animal experiment, no association with PCa, and experiment on DNA microarray were the reasons for excluding these articles. The full-text articles were evaluated for the remaining 5 studies, and all of them (6 datasets) were retained in the final meta-analysis. These miRNA data sets were obtained from the National Centers for Biotechnology Information (NCBI) Gene Expression Omnibus (GEO) database (http://www.ncbi.nlm.nih.gov/geo/).

### The microRNA datasets and individual data analysis

In this study, a total of six microRNA datasets related to the recurrent PCa after RP (GSE55323 [10], GSE26245 and GSE26247 [11], GSE65061 [17], GSE62610 [12], and GSE46738 [14]) met the inclusion criteria and were selected for meta-analysis.

In the GSE55323, a total of 41 recurrent and 41 non-recurrent tumors after RP, which have been obtained from Baylor College of Medicine Prostate Cancer program, have been considered for performing miRNA profiling. Recurrence has been defined as a two consecutive serum PSAs greater than 0.2 ng/ml. To carry out microarray analysis, 20 samples from each group have been profiled using miRNA microarray chips.

In GSE26245 and GSE26247, total RNA from 71 formalin-fixed-paraffin-embedded (FFPE) specimens with known long-term outcome have been used for performing DASL expression profiling with a custom-designed panel of 522 PCa relevant genes. Recurrence has been defined as a two consecutive serum PSAs greater than 0.2 ng/ml. In the GSE26245, samples from 71 patients (29 with BCR and 42 without BCR) and in the GSE26247, samples from 82 patients (29 with BCR and 53 without BCR) have been used. In this study, the samples with unknown BCR have been removed.

For the GSE65061, total RNA has been extracted from tumor-enriched 1mm cores from 43 RP paraffin tissue blocks. Tissue isolated at the time of RP has been utilized for miRNA profiling. Thirty-six months has been considered as the cutoff, as it was near the median time to recurrence. From 43 patients, 19 were labeled as the samples with BCR (≤ 36 months) and 24 as the samples without BCR (> 36 months).

In the GSE62610, total RNA has been taken from tumor-enriched 1.5 mm cores in diameter from 36 formalin fixed paraffin embedded (FFPE) specimens. Then biochemical failure has been defined as two consecutive measurements of PSA > 0.2 ng/ml. From 36 patients 22 has been classified as the samples with BCR and 14 as the samples without BCR. In the GSE62610 most of microRNAs have null expression. After excluding miRNAs with no expression in any of the samples, 536 miRNAs have been kept for further analysis.

For GSE46738, total RNA has been taken from tumors from the 51 patients that underwent an RP by the same surgeon to treat localized PCa. In the GSE46738, the BCR status of samples is not mentioned explicitly. In the present study, according to the expression level of the miRNAs with greater statistical power, which has been reported in the third table of the study [14], tumors were divided into the positive BCR and the negative BCR by using clustering techniques. In GSE46738, from 51 samples, 34 were classified as the samples with BCR and 17 as the samples without BCR. Table 1 has provided detailed information of each dataset.

**Table 1:** Datasets used in the meta-analysis.

Study Set	GEO Accession	Platform of dataset	Type of Platform	#of samples (BCR+, BCR−)*	# of miRNAs	References	Model for generating expression summaries
1	GSE55323	GPL10701	Agilent-021827 Unrestricted Human 15.7K v3.0 miRNA Microarray	40 (20, 20)	15744	[10]	log2 transformed and quantile normalized
2	GSE26245	GPL11350	Illumina Custom Prostate Cancer DASL Panel miRNA	71 (29, 42)	733	[11]	quantile-normalized expression signal
	GSE26247	GPL11350	Illumina Custom Prostate Cancer DASL Panel miRNA	82 (29, 53)	1145	[11]	quantile-normalized expression signal
3	GSE65061	GPL17537	nCounter Human miRNA Expression Assay, V2	43 (19, 24)	800	[17]	normalized data
4	GSE62610	GPL18942	Applied Biosystems Taqman Low Density Array Human microRNA Card A+B Set v3.0	36 (22, 14)	536	[12]	normalized data
5	GSE46738	GPL8786	[miRNA-1_0] Affymetrix miRNA Array	51 (34, 17)	847	[14]	log scale RMA generated

*BCR+/−, biochemical disease recurrence status after RP (positive, negative).

The microRNA microarray datasets were obtained from GEO NCBI. All GEO series matrix files (GSE), platform sets, and annotation files were downloaded and parsed using ‘GEOquery’ package of Bioconductor 3.2 in R version 3.2.2. To identify Differentially Expressed (DE) miRNAs in each individual dataset, moderated t-test was used.

### Microarray meta-analysis

This meta-analysis was performed in accordance with the guidelines provided in [18]. First, each individual dataset was preprocessed using the log2 transformation and normalization. Then, any training and validation dataset (GSE26245 and GSE26247) was combined together. Next, gene matching was done for all microRNA probes. When multiple probe sets matched to an identical gene symbol, the probe that presented the greatest inter-quartile range (IQR) was selected to represent the target gene symbol. After matching all probes to a common microRNA gene symbol, differential expression analysis was performed with “MetaDE” package for each dataset independently using adjusted p-value < 0.05, based on the false discovery rate by the Benjamini–Hochberg procedure [19] and moderated t-test.

Data integrity was checked for all datasets, and the differential expression meta-analysis across recurrence and non-recurrence samples was carried out by p-values combination using Adaptive Weight (AW) and Fisher methods.

### Statistical analysis

The meta-analysis was performed using the ‘MetaDE’ package in R [20]. The moderated t-statistic was utilized to identify DE miRNAs in each individual dataset. The Fisher and AW were used to combine the p-values from moderated t-test for meta-analysis. Fisher’s method is a summation of–log (p-value) across studies. An adjusted p-value of < 0.05, based on the False Discovery Rate (FDR) using the Benjamini–Hochberg procedure was used to select DE microRNA genes.

### Network analysis of common differentially expressed microRNA genes

Network-based analysis was performed using a MIROB web tool (http://mirob.interactome.ru/) which has been designed to support analysis of microRNA expression data. MIROB (microRNA OncoBase) scans the set of input miRNAs to build any cancer pathways, detects key targets and Transcription Factors (TF) of candidate miRNAs, identifies any possible correlation between key targets and TF of candidate miRNAs and other diseases, and makes pathogenesis network.

### Functional gene set enrichment analysis of common differentially expressed microRNA genes

The TF and target genes of DE miRNAs were searched for the pathways in which they participate, using the EnrichR web-tool [21]. Specifically, Gene Ontology (GO) analysis, Kyoto Encyclopedia of Genes and Genomes (KEGG) pathway analysis, and Reactome pathway analysis were performed. Moreover, in order to find metabolic pathways and biochemical reactions, pathway commons analysis was performed for candidate DE miRNAs using a pathway commons network visualizer (PCViz) web tool. To further investigate the function of the DE miRNAs, they were also mapped to the most significant KEGG pathway.

### Diagnostic performance of common differentially expressed microRNA genes

Based on the hypothesis that miRNA classifiers may improve sensitivity and specificity over single markers, the diagnostic potential of 37 DE miRNAs classifier was trained and tested in each dataset by performing receiver operating characteristic (ROC) analysis using the normalized expression values of miRNA genes. Logistic regression classifier with leave-one-out cross validation (LOOCV) scheme was used for this analysis. To show the discriminatory power of 37 DE miRNAs for distinguishing recurrent PCa samples from non-recurrent samples, area under the ROC curve (AUC) was calculated using WEKA open source machine learning software. Furthermore, the best subset of DE microRNAs which improves the BCR prediction over original studies and 37 DE miRNAs set, was identified in each dataset by using geometric particle swarm optimization (PSO). The LOOCV AUC of logistic regression was utilized as the fitness function. Partial C4.5 decision tree (PART) was used to find the relation and rules between the DE microRNAs in each dataset for biological point of view.

## Results

### Identification of common differentially expressed microRNAs for prostate cancer recurrence by meta-analysis

**Fig 1:**
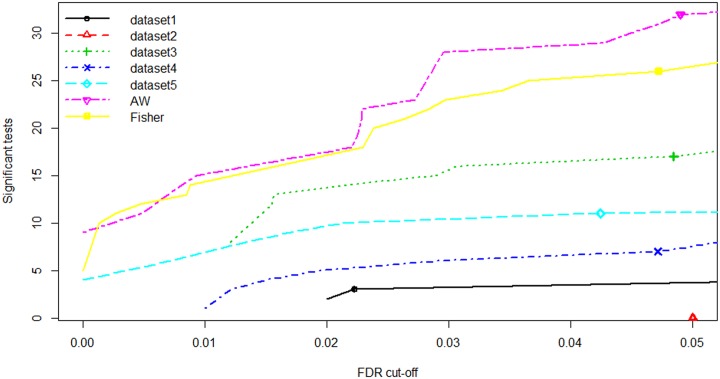
P-value (or FDR) vs number of detected miRNAs for individual analysis as well as meta-analysis. In each individual dataset, moderated-t statistics was used to generate p-values while adaptive weight and Fisher's methods were utilized to combine these p-values for meta-analysis. This figure is generated using the “MetaDE” package in R.

**Fig 2:**
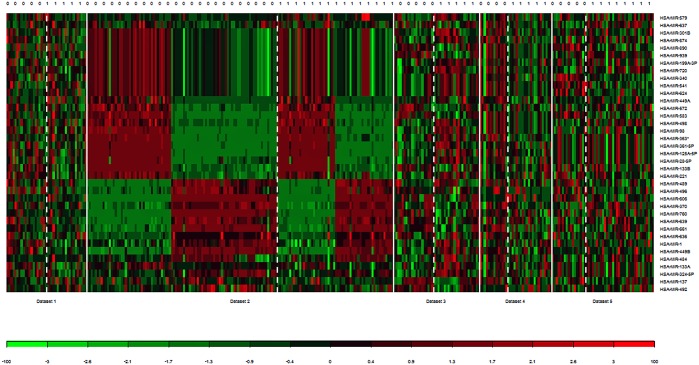
The heat map of the actual expression profiles for the 15 up- and 22 downregulated DE microRNAs obtained from the meta-analysis. The heat map is generated using the “MetaDE” package in R. The expression profiles greater than the mean are colored in red and those below the mean are colored in green. 0: Non-recurrence; 1: Recurrence.

To identify a common DE microRNAs for PCa recurrence, five miRNA studies (Table 1) were analyzed using “MetaDE” package in R. First, individual analysis was performed and the moderated t—test was used to calculate the p-values which frequently used in meta-analysis. Then, AW and Fisher's method were utilized to combine the p-values and find miRNAs that were differentially expressed between samples with recurrence and non- recurrence (+/− BCR) across all studies. From miRNA microarray meta-analysis, we identified a total of 37 DE miRNAs including 15 overexpressed and 22 under expressed microRNAs across at least two datasets under the significance threshold of adjusted p-value < 0.05. Fig 1 shows the number of DE microRNAs against FDR obtained from individual analysis as well as meta-analysis. It is clearly seen that the meta-analysis has detected more candidate markers. Fig 2 shows the heat map of those 37 microRNAs. A complete list of DE microRNAs has been provided in Table 2. The miR-449A, miR-484, and miR-579 were among the most significant overexpressed genes, while miR-449B, miR-1, miR-137, miR-370, miR-375 were the most under expressed genes across all miRNA datasets (See Table 2).

**Table 2:** The 37 shared significantly deregulated miRNAs identified in the meta-analysis.

**down regulated**	**GSE55323**		**GSE26245, GSE26247**		**GSE65061**		**GSE62610**		**GSE46738**		**Merged data**		
	**P-value**	**FC**	**P-value**	**FC**	**P-value**	**FC**	**P-value**	**FC**	**P-value**	**FC**	**Meta. Stat**	**Meta. P value**	**Meta. FDR**
miR-1	0.0039	-1.77	0.0872	-1.2	0.0125	1.72	0.799	-1.08	0.7681	1.07	25.6944	0.0039	0.0342
miR-133A	0.0256	-1.19	0.01529	-1.22	0.02678	1.62	0.5653	-1.17	0.1863	1.35	24.066	0.00245	0.040833
miR-133B	0.0041	-1.41	0.3924	1.1	0.0188	-1.23	0.9675	-1.01	0.5129	1.16	22.2085	0.0089	0.0294
miR-137	0.3072	1.08	0.032	-1.18	0.0091	-2.69	0.0129	-11.49	0.1852	-1.08	30.7251	0.0005	0.019
miR-221	0.00065	-1.51	0.7294	1.03	5.40E-05	2.33	0.506	1.22	0.099	-1.29	19.26	0.00074	0.0477
miR-340	0.8665	1.01	0.8959	1	0.6935	-1.04	0.31227	-1.31	<0.001	-1.89	20.7435	0.00044	0.04
miR-370	0.395	-1.12	0.1995	-1.26	0.1671	-1.3	0.0422	-2.15	0.0037	1.85	26.1757	0.0033	0.0342
miR-449B	0.0485	-1.2	0.2516	-1.14	NA	NA	0.00318	-3.97	0.0676	1.39	22.501	0.000381	0.044
miR-489	0.2688	-1.08	0.9699	-1	0.6031	1.04	0.0164	-1.67	0.0179	-1.54	19.9117	0.0074	0.0455
miR-492	0.8683	1.11	0.0269	-1.07	0.0001	-1.4	0.8009	-7.16	0.7347	1.09	26.547	0.0012	0.025
miR-496	0.001	-1.69	0.1743	-1.17	0.6391	-1.05	0.04696	-3.48	0.05464	-1.28	26.5035	0.0003	0.008
miR-541	0.4518	1.06	NA	NA	0.0042	-1.51	NA	NA	<0.001	-1.69	21.215	0.00032	0.05
miR-572	0.2212	-1.21	0.2326	1.08	0.005	-1.31	0.0531	-1.41	0.3638	1.25	24.4192	0.00446	0.0416
miR-583	0.5071	1.07	0.442	1.1	0.0089	-1.42	NA	NA	0.00061	-1.51	27.205	0.00061	0.048
miR-606	0.3955	1.09	0.2885	-1.21	0.1067	-1.22	0.9715	-1.03	0.001	-1.78	22.7104	0.0042	0.05
miR-624	0.1552	1.12	0.6498	1.07	0.05	-1.26	0.0296	-1.98	0.0002	-1.48	20.58	0.00039	0.038
miR-636	0.6497	1.03	0.4884	1.05	0.8496	-1.03	<0.001	-2.06	0.5493	-1.09	95.9233	<0.001	<0.001
miR-639	0.004	-1.16	0.85501	1.04	0.2339	-1.16	0.3246	-1.2	0.1879	1.13	19.5331	0.0082	0.0455
miR-661	0.9746	1	0.11	-1.11	0.04517	-1.29	0.00053	-1.32	0.27696	1.06	23.87	0.00125	0.028
miR-760	0.4702	1.13	0.2285	-1.21	0.0003	-1.46	0.2971	-1.3	0.1529	1.19	24.3088	0.0022	0.035
miR-890	0.489	-1.14	NA	NA	0.0442	-1.24	NA	NA	0.0002	-1.86	23.07	0.00014	0.013
miR-939	0.8377	1.03	NA	NA	0.0085	-1.32	NA	NA	0.0288	1.46	16.61	0.0023	0.049
**Up regulated**	**P-value**	**FC**	**P-value**	**FC**	**P-value**	**FC**	**P-value**	**FC**	**P-value**	**FC**	**Meta. Stat**	**Meta. P value**	**Meta. FDR**
miR-125A-5P	0.24	-1.13	0.632	1.17	0.0011	1.58	0.06081	-1.46	NA	NA	22.9155	0.0028	0.038
miR-199A-3P	0.7639	-1.05	NA	NA	0.00172	1.78	0.344	-1.3	NA	NA	0.0016	0.00274	0.042
miR-28-5P	0.6761	-1.05	0.9039	-1.02	0.00041	1.47	0.3982	-1.22	NA	NA	24.05	0.0024	0.04
miR-301B	0.7513	-1.01	NA	NA	0.0049	1.59	0.0164	-1.76	0.717	-1.02	20.0917	0.0066	0.0455
miR-324-5P	0.147	-1.13	0.1263	-1.13	0.0001	1.55	0.6594	-1.13	NA	NA	32.2291	0.00065	0.01625
miR-361-5P	0.3474	-1.08	0.3478	1.21	0.00083	1.76	0.5897	-1.14	NA	NA	0.00077	0.00122	0.038
miR-363*	0.1773	1.14	0.2258	1.41	NA	NA	0.00038	-1.95	NA	NA	22.176	0.0005	0.044
miR-449A	0.0332	1.35	0.5059	1.08	0.6952	1.05	0.0007	-5.43	0.2048	1.38	26.5308	0.0031	0.0342
miR-484	0.152	1.3	0.2685	1.09	0.0049	1.19	0.1188	-1.42	0.1252	1.33	25.4578	0.0043	0.0342
miR-498	0.7157	1.03	0.2734	1.08	0.0147	1.37	NA	NA	0.0013	1.66	25.0151	0.0019	0.035
miR-579	0.1908	-1.1	<0.001	1.38	0.0338	1.23	0.6918	-1.16	0.8592	1.01	29.3	0.00025	0.01625
miR-637	0.5443	1.07	0.6948	1.01	0.2487	-1.17	NA	NA	0.0001	2.22	20.69	0.00055	0.0375
miR-720	0.0175	-1.69	NA	NA	0.0008	1.76	NA	NA	0.0043	1.96	25.13	7.30E-05	0.0125
miR-874	0.1547	-1.18	NA	NA	0.00321	1.55	0.00017	-2.05	0.30732	1.29	34.7751	0.000178	0.0208
miR-98	0.80911	-1.02	0.6266	1.07	0.00016	1.77	NA	NA	0.8112	1.03	23.903	0.0007	0.04625

The “moderated t-test” is used to perform individual analysis and calculate p-values. The corresponding p-values are adjusted, based on the false discovery rate using the Benjamini–Hochberg procedure used to select DE miRNAs across at least two datasets.

“*”, denotes the mature miRNA sequence.

‘‘NA”, represents ‘‘not available”.

### Identification of the TF and regulatory network for the differentially expressed microRNAs obtained from meta-analysis

**Fig 3:**
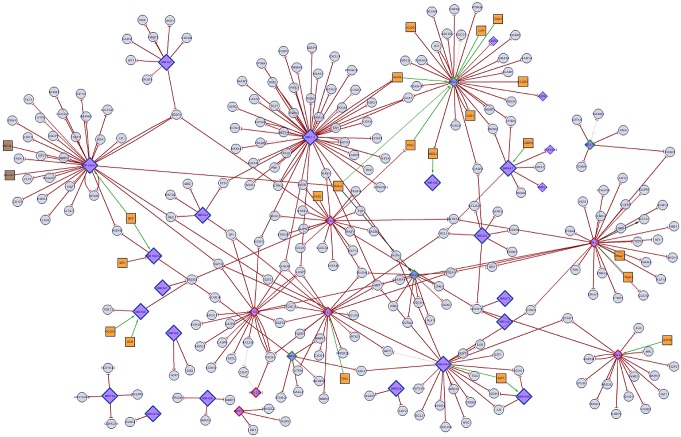
Network interrelation of DE microRNAs identified in the meta-analysis. Orange squares show TF. The circles show the targets of DE microRNAs. Green and red lozenges show up regulated and down regulated microRNAs in various types of diseases. The network was generated using a MIROB web tool to explore DE microRNAs relationships and collective functions.

MIROB tool was used to perform regulatory microRNA network analysis to identify regulators responsible for the observed patterns in miRNA meta-analysis studies. The interaction network was constructed between DE microRNAs, TF and target genes associated with the complete set of DE (Fig 3). Twenty four of DE miRNAs were found in the network. The details of those miRNA gene networks have been given in Table 3. Key targets, ontology information on target genes, TF and a descriptive analysis of expression of the DE miRNAs have been summarized in this table. In addition, it shows that DE miRNAs are highly associated with colorectal, PCa, breast, and gastric cancer.

**Table 3:** The details of 37 DE miRNAs that are involved in the interaction network, which has been drawn by MIROB.

MicroRNAs	Transcription Factors	Target genes	Disease influence (expression)	pathogenesis of a disease
miR-1	SNAI2	FOXP1, HDAC4, PDLIM5, PIM1, CCND2, CXCL12, PNP, LASP1, SNAI2, PAX7, KLF4, MET, FN1, PTMA, TAGLN2, PAX3, GJA1, SOX6, ATP6V1B2, LARP4, CNN3, HSPD1, HSPA4, POGK, PGM2, SERP1, NETO2, Srxn1, CAND1, ADAR, KIF2A, G6PD, MEF2A, KCNJ2, PPP2R5A, HCN2, TWF1, HCN4, KCNE1, ANXA2, ETS1	-	Metastasis, Angiogenesis, growth, Proliferation, Invasion, migration, Apoptosis, cell cycle arrest, differentiation, WNT signaling.
miR-125A	NFATC1, TP53	RHOA, FYN, CDKN1A, EDN1, BAK1, ARID3B, CD34, ERBB2, ERBB3, NTRK3, ELAVL1, TNFAIP3, PDPN, KLF13, CLEC5A, TRAF6, RAF1, ZBTB7A, VEGFA	Colorectal cancer (down)	Proliferation, Invasion, migration, differentiation, cell cycle arrest, Angiogenesis, survival, Sorafenib resistance, myeloid, differentiation
miR-133A	-	CD47, LASP1, GSTP1, FSCN1, ARPC5, TAGLN2, CASP9, KCNH2, CACNA1C, HCN2, KCNQ1, EGFR, IGF1R, RFFL, SP1, ABCC1, FOXC1, BCL2L1	Prostate Cancer (down)	Proliferation, Invasion, migration, Apoptosis, cell cycle arrest, colony formation, ERK pathway (MAPK pathway), Liver metastasis, Lung metastasis, tumor growth, Adriamycin (Adr) resistance, 5-fluorouracil resistance, cisplatin resistance
miR-133B	TP63	BCL2L2, MCL1, FGFR1, FSCN1, MET, PITX3, IGF1R, CXCR4, UTRN, SP1, RHOA, MMP9, EGFR, TAGLN2, LASP1, SIRT1, PPP2R2D, FOXC1, PTBP1	Colorectal cancer (up), Prostate Cancer (down), Gastric (down)	Proliferation, Invasion, migration, Apoptosis, cell cycle arrest, WNT signaling, tumor growth, cisplatin resistance, Cell growth
miR-137	FOXD3, HMGA1	CDK6, CDC42, SLC7A1, KDM1A, CSMD1, C10orf26, CACNA1C, TCF4, ESRRA, CTBP1, FMNL2, MIB1, GLIPR1, CSE1L, PTGS2, MITF, PXN, PTBP1, NF1, EPHA7, AKT2, ZBTB7A, HEY2, KLF12, MYO1C, CUL4A, FOXO1, CDK6.	Colorectal cancer (down), Gastric (down)	Metastasis, Angiogenesis, growth, colonyformation, Proliferation, Invasion, migration, Apoptosis, tumorgrowth, Cellgrowth, cellcyclearrest, Stemness, cell viability, aerobic glycolysis, cell cycle
miR-199A1	SRF, SPI1, SNHG12, SNHG1, RELA	ST6GAL1, HSPA5, ATF6, ERN1, IKBKB, CACUL1, CAV2, MTOR, LIF, RELA, NFKB1, ATG7, CLTC, NLK, CDH1, SLC27A1, MAP4K3, CD151, YAP1, OSCP1, HIF1A, VEGFA, IGF1R, IGF2, FLT1, KDR, HGF, MMP2, E2F3, ACVR1B	-	Proliferation, Invasion, migration, Apoptosis, cell cycle arrest, Angiogenesis, colony formation, ERK pathway (MAPK pathway), tumor growth, cisplatin resistance, cell viability, Chemoresistance, survival, Sorafenib resistance, Autophagy, adhesion
miR-221	FOSL1, SNAI2, RELA, JUN, ESR1, NCOR2, NCOR1, TP53	CERS2, TRPS1, DICER1, KIT, NOS3, BBC3, MBD2, CDKN1C, GJA1, ICAM1, CDKN1B, DIRAS3, RAB1A, HECTD2, TICAM1, PTPRM, MGMT, FOXO3, RECK, MDM2, PTEN, SOCS1, CASP3	Breast cancer (up), Colorectal cancer (up), Gastric (up)	Proliferation, Invasion, migration, Apoptosis, cell cycle arrest, Metastasis, Cell growth, motility, cell cycle progression, Chemoresistance, doxorubicin resistance, Radioresistance, survival, Sorafenib resistance
miR-28	STAT5B	STAT5B, CDKN1A, CCND1, HOXB3, NME1, N4BP1, OTUB1, TEX261, MAPK1, E2F6, MPL, BAG1, MAD2L1, RAP1B, IL34, IGF1	Colorectal cancer (down)	Proliferation, Invasion, migration, Apoptosis, cell cycle arrest, Metastasis, ERK pathway (MAPK pathway), P38 signaling, AKT signalling, PI3K signaling
miR-301B	-	FOXF2	-	-
miR-324	-	SMO, GLI1, WNT2B, ETS1, SP1	-	Proliferation, Invasion, migration, cell cycle arrest, Metastasis, Radioresistance
miR-340	RELA	RELA, MET, ROCK1, PTBP1, SOX2, MITF, RHOA, PLAT, DMD, JAK1, CCNG2	Gastric (up)	Proliferation, Invasion, migration, differentiation, cell cycle arrest, Metastasis, tumor growth, Cell growth, stemness, aerobic glycolysis, cell viability, cell cycle progression, Senescence, JAK/STAT signaling
miR-361	-	STAT6, VEGFA, TWIST1, WT1, SH2B1, CXCR6, SND1, PHB	-	Proliferation, Invasion, migration, Apoptosis, Metastasis, colony formation, tumor growth, Cell growth, stemness
miR-363	-	CDKN1A, S1PR1, BCL2L11, CASP3, CD276, FBXW7, MCL1	-	Proliferation, Apoptosis, cisplatin resistance, cell viability, Chemoresistance, survival
miR-370	-	CPT1A, TGFBR2, FOXM1, FOXO1, ENG	Gastric (up)	colony formation, Proliferation, Apoptosis Chemoresistance, colony formation, cisplatin, resistance
miR-449A	E2F1, EZH2, MYCN	E2F3, CDC25A, MET, SIRT1, CDK6, BCL2, CCND1, CRHR1, LEF1, KLF4, NOTCH1, HDAC1, AR, IL6R, SOX4, CREB5, FOS, MYC	Prostate Cancer (down), Gastric (down)	Metastasis colony, formation, Proliferation, Invasion, migration, Apoptosis, motility, EMT, cell cycle arrest, cisplatin resistance, differentiation, Cell growth, cell viability, Radioresistance, Senescence, Antiapoptosis
miR-449B	E2F1, AR	CDK6 CDC25A, HDAC1, SOX4	-	Proliferation, migration Apoptosis, Cell growth colony formation, cell viability
miR-489	-	SMAD3, MMP7, PROX1	-	Proliferation, Invasion, migration, Lung metastasis, Adriamycin (Adr) resistance, EMT
miR-492	-	BSG, SOX7	-	Proliferation, Oxaliplatin, resistance
miR-498	VDR, NCOA3	TERT, ERBB2	-	Apoptosis, tumor growth, Cell growth
miR-661	CEBPA	STARD10, PVRL1, MTA1, MCL1, MDM2, MDM4, PTEN	-	Proliferation, Invasion, migration, cell cycle arrest, Metastasis, tumor growth, motility, EMT
miR-760	-	CSNK2A1, HIST1H3D, HIST1H2AD, PHLPP2	-	Proliferation, colony formation, Senescence
miR-874	-	AQP3, PIN1, MAGEC2	Gastric (down)	Proliferation, Invasion, Apoptosis, colony formation, Cell growth, mTOR signaling
miR-939	-	APC2, NGFR	-	Proliferation, WNT signaling
miR-98	EZH2	ACVR1B, MMP11, EZH2, SALL4, IGF2BP1, CTHRC1	Gastric (up)	Angiogenesis, growth, Proliferation, Invasion, migration, Apoptosis, EMT, cell cycle arrest, WNT signaling,

### Further enrichment analysis for identification of overrepresented biological pathways and gene ontology terms

We performed gene set enrichment analysis by EnrichR tool, using the complete list of key targets and TF of DE miRNAs. GO terms and biological pathways were significantly overrepresented in the gene list if they showed an adjusted p-value < 0.05. Results for gene ontology and enriched biological pathways (KEGG, Reactome) have been shown in Tables 4–6, respectively. DE microRNAs in meta-analysis results were associated with the enriched pathways with adjusted p-value < 0.05, including “MicroRNAs in cancer (hsa05206)”, “Pathways in cancer (hsa05200)”, “Proteoglycans in cancer (hsa05205)”, “PI3K-Akt signaling pathway (hsa04151)”, “Prostate cancer (hsa05215)” and “Signal Transduction (R-HSA-162582)”. The most important GO terms associated with key targets and TF of DE miRNA genes included “regulation of epithelial cell proliferation (GO: 0050678)”, “tissue morphogenesis (GO: 0048729)”, “regulation of cellular response to stress (GO: 0080135)”, and “positive regulation of cellular component movement (GO: 0051272)”.

**Table 4:** Top enriched gene ontology (GO) biological process identified by functional analysis of the target genes and TFs of the DE microRNAs in the meta-analysis.

GO-ID	Description	Overlap*	Adjusted P-value
GO:0050678	regulation of epithelial cell proliferation	32/258	7.430E-18
GO:0048729	tissue morphogenesis	35/358	1.191E-16
GO:0080135	regulation of cellular response to stress	36/404	4.884E-16
GO:0051272	positive regulation of cellular component movement	31/296	1.209E-15
GO:0070482	response to oxygen levels	29/259	2.118E-15
GO:2001233	regulation of apoptotic signaling pathway	33/356	2.466E-15
GO:2000147	positive regulation of cell motility	30/287	2.699E-15
GO:0040017	positive regulation of locomotion	30/304	1.184E-14

Gene sets functional analysis was performed using extended libraries of the EnrichR tool.

* Overlap: indicates the number of hits from the meta-analysis compared to each curated gene set library.

**Table 5:** Top enriched KEGG pathways identified by functional analysis of the target genes and TFs of the DE microRNAs in the meta-analysis.

Pathway ID	Name	Overlap*	Adjusted P-value
hsa05206	MicroRNAs in cancer	56/297	3.476E-45
hsa05200	Pathways in cancer	55/397	4.152E-37
hsa05205	Proteoglycans in cancer	33/203	4.675E-24
hsa04151	PI3K-Akt signalling pathway	38/341	7.293E-22
hsa05215	Prostate cancer	23/89	1.259E-21
hsa05212	Pancreatic cancer	20/66	2.252E-20
hsa05218	Melanoma	19/71	2.982E-18
hsa05220	Chronic myeloid leukemia	19/73	4.676E-18
hsa04520	Adherens junction	19/74	5.524E-18
hsa04933	AGE-RAGE signalling pathway in diabetic complications	21/101	7.221E-18

Gene sets functional analysis was performed using extended libraries of the EnrichR tool.

* Overlap: indicates the number of hits from the meta-analysis compared to each curated gene set library.

**Table 6:** Top enriched reactome pathways identified by functional analysis of the target genes and TFs of the DE microRNAs in the meta-analysis.

Pathway ID	Name	Overlap*	Adjusted P-value
R-HSA-162582	Signal Transduction	100/2465	4.220E-22
R-HSA-1266738	Developmental Biology	46/786	2.574E-14
R-HSA-1236394	Signalling by ERBB4	29/330	5.348E-13
R-HSA-166520	Signalling by NGF	33/450	8.395E-13
R-HSA-180292	GAB1 signalosome	19/125	1.065E-12
R-HSA-198203	PI3K/AKT activation	19/125	1.065E-12
R-HSA-5654695	PI-3K cascade:FGFR2	18/122	4.702E-12
R-HSA-1257604	PIP3 activates AKT signalling	18/122	4.702E-12

Gene sets functional analysis was performed using extended libraries of the EnrichR tool.

* Overlap: indicates the number of hits from the meta-analysis compared to each curated gene set library.

**Fig 4:**
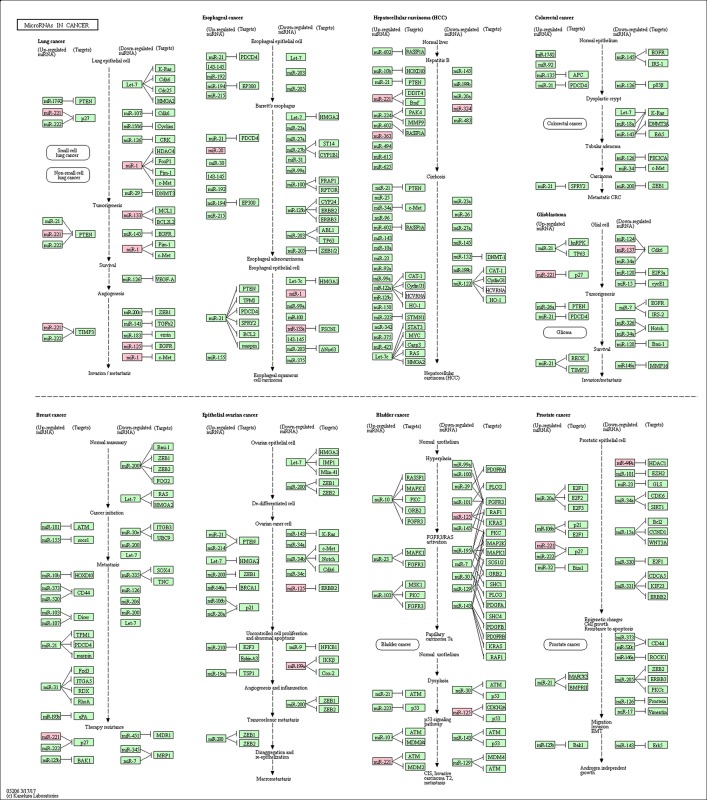
The most significant enriched KEGG pathway for the DE microRNAs identified from meta-analysis. The microRNAs in the red box indicates co-deregulated microRNA genes in our list. The DE microRNAs identified from meta-analysis were mapped to “microRNAs in cancer” pathway (KEGG-ID: hsa05206) by using the KEGG mapper web tool.

**Fig 5:**
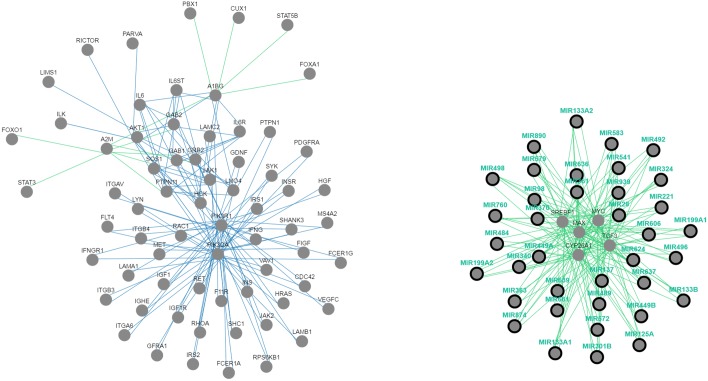
Common pathway analysis for DE microRNAs identified from meta-analysis. This analysis revealed that TCF3, MYC, MAX, CYP26A1 and SREBF1 are significantly interacting with candidate miRNA genes.

To further investigate the function of DE miRNAs, we mapped them to the KEGG database. Eleven of them (miR-1, miR-125A, miR-133A, miR-133B, miR-137, miR-199A, miR-221, miR-28, miR-324, miR-363 and miR-449A) were found in the “miRNAs in cancer” pathway (KEGG-ID: hsa05206; Fig 4) with adjusted *P*-value of 7.554e-15 (Table 5). Moreover, common pathway analysis revealed that TCF3, MYC, MAX, CYP26A1, and SREBF1 significantly interact with DE miRNAs (Fig 5).

### Diagnostic performance

**Fig 6:**
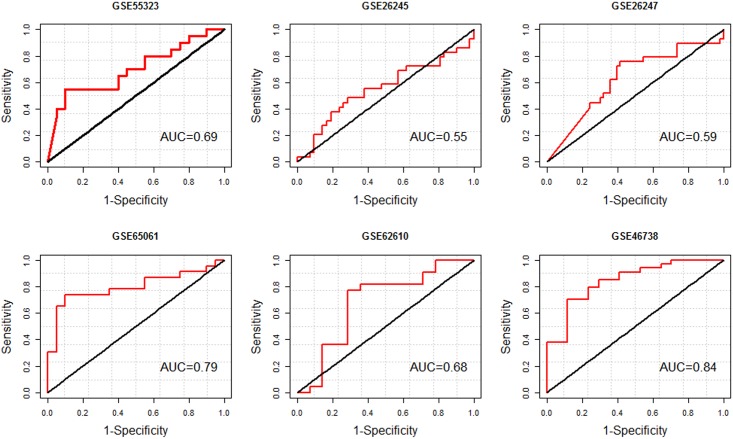
Receiver operating characteristics (ROC) analysis of 37-miRNA signature in biochemical disease recurrence vs. the non-recurrence samples using each GEO datasets. The DE miRNAs are depicted in Table 2. AUC; area under the ROC curve.

We assessed the diagnostic potential of the 37-miRNA signature identified by meta-analysis. ROC curve analysis gave AUCs from 0.55–0.84 for miRNAs set in each GEO dataset (See Fig 6). To investigate whether a miRNA signature may increase diagnostic accuracy over 37-miRNA signature, we employed a soft computing technique (PSO/ logistic regression) and trained and tested on miRNA expression profiles. The best subset of DE miRNAs was identified in each GEO dataset and shown in Table 7.

**Table 7:** Best subset, PART’s decision rules and diagnostic potentials for the DE microRNAs identified from meta-analysis in 6 GEO datasets.

GEO Accession	Best subset	Extracted rules by PART	PART’s AUC (95%CI^↑^)	PART’s F-measure
**GSE55323**	miR-1, miR-221, miR-28-5P, miR-301B, miR-324-5P,miR-370,miR-449A, miR-606, miR-624, miR-661, miR-98	**IF** miR-496 > 8.13 **AND** mir-1 >9.67 **AND**: BCR- (11.0) **IF** miR-137 > 6.36 **AND** miR-449A ≤ 7.10 **AND** mir-137 ≤ 6.82: BCR- (11.0/2.0)	0.75	0.72
**GSE26245**	miR-370, miR-492, miR-579, miR-639, miR-98	**IF** miR-579 ≤ 9.401: **AND** miR-639 ≤ 9.120: BCR- (46.0/10.0) **IF** miR-324-5P > 12.032: BCR+ (17.0/1.0) **IF** mir-639 >9.212: BCR- (5.0)	0.60	0.78
**GSE26247**	miR-1, miR-133A, miR-137, miR-363*	**IF** miR-363* ≤ 8.89 **AND** miR-636 ≤ 9.34: BCR- (43.0/4.0) **IF** miR-363* > 8.44 **AND** miR-661 > 12.95: BCR+ (20.0)	0.804	824
**GSE65061**	miR-1, miR-221-3P, miR-301B, miR-489, miR-637, miR-939,miR-98	**IF** miR-221-3P ≤ 6.97 **AND** miR-489 > 5.36 **AND** miR-98 ≤ 7.84 **AND** miR-939> 3.87 **AND** miR-637 ≤ 4.17: BCR- (14.0) **IF** miR-301B > 3.97 **AND** miR-221-3P > 6.029: BCR+ (21.0) **IF** miR-1 > 5.23: BCR- (6.0)	0.734	0.744
**GSE62610**	miR-449A, miR-496, miR-636, miR-492	**IF** miR-449A > 16.80 **AND** miR-636 ≤ 17.73 **AND** miR-496 ≤ 20.304: BCR+ (12.0/2.0) **IF** miR-449A **>**16.804: BCR- (13.0/1.0)	0.763	0.833
**GSE46738**	miR-340,miR-541, miR-624	**IF** miR-340 ≤ 2.13 **AND** miR-541 ≤ 3.107: BCR+ (33.0/1.0) **IF** miR-541 > 1.374: BCR- (16.0)	0.8823	0.865

^↑^ CI: confidence interval.

**Fig 7:**
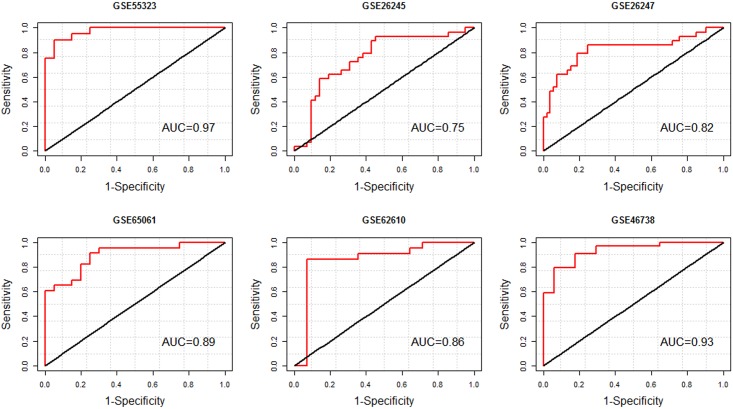
ROC analysis of the best subset of the DE miRNAs in biochemical disease recurrence vs. the non-recurrence samples using each GEO datasets. The best subset of DE miRNAs is shown in the first column of Table 3 which has been found by using soft computing technique (PSO/ logistic regression).

Notably, the discriminating power of the identified signatures in each GEO dataset is higher than the case where 37-miRNA classifier was considered. For the best subset of DE miRNAs in each GEO dataset, the ROC curve analysis gave AUCs from 0.75–0.97 (See Fig 7). The highest diagnostic accuracy (97%) was given for GSE55323 with 11-miRNAs. Moreover, in order to correctly classify BCR+ vs. BCR- samples, simple rules were extracted using a decision tree classifier (Table 7). Among six GEO datasets, rules with high diagnostic potentials were extracted for GSE46738 and GSE26247.

**Fig 8:**
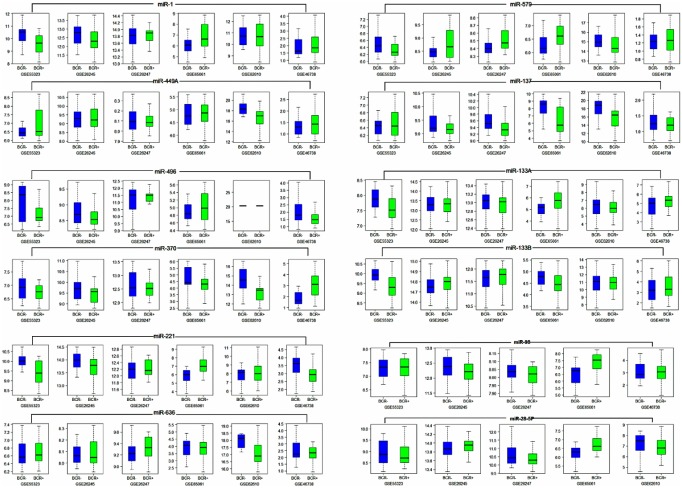
A comparison between expression of co-deregulated microRNAs in recurrent vs. non-recurrent PCa samples. Those miRNAs that were selected for analysis are depicted above the box plots (Table 3). Lines within the boxes indicate median values; whiskers—min and max for miRNA values. BCR+/ -, biochemical disease recurrence status (positive, negative).

Finally, a comparison between the expressions of co-deregulated microRNAs in BCR+ vs. BCR- was done by plotting boxplots (Fig 8). The boxplots were drawn for co-deregulated microRNAs that are involved in the PCa pathway.

## Discussion

Various miRNAs are DE in individuals with recurrent PCa, and identifying the most important miRNAs and pathways associated with the disease is very important. A meta-analysis of multiple miRNA datasets combines the generated p-values of individual studies, making the identification of DE microRNA genes more reliable.

In this study, we attempted to identify common miRNAs underlying recurrent PCa using meta-analysis of six publicly available microRNA datasets to focus deeply on identifying DE microRNA genes and risk factors shared between them.

By meta-analysis of six published miRNA expression datasets of recurrent PCa, we identified a common signature of a total of 37 DE microRNAs including 15 overexpressed and 22 under expressed microRNA genes across at least two datasets under the significance threshold of adjusted p-value < 0.05 in recurrence compared to non-recurrence samples. The identified 37 microRNAs in this meta-analysis were discovered as DE microRNAs in at least one dataset in the prior individual analysis. Of the 37 DE miRNAs associated with BCR after RP (Table 2), all except miR-606 have been reported to be associated with cancer in general [22–35]. Fifteen miRNAs (miR-1, miR-133A, miR-133B, miR-449A, miR-137, miR-370, miR-221, miR-449B, miR-125A-5P, miR-199A-3P, miR-301B, miR-340, miR-361, miR-363, miR-98) have been previously linked to PCa [36–46] and of those, miR-1, miR-133B, miR-449B, and miR-221 have been described as predictive markers in PCa recurrence after RP [10, 12,13].

Among the overexpressed DE microRNAs, miR-449A and miR-579 had high combined P-values across all studies.

Tumor-suppressive miR-449A targets HDAC1 and induces growth arrest in PCa [37]. It also causes Rb-dependent cell cycle arrest and senescence in PCa cells (Table 3) [47]. For a previously poorly characterized miRNA, namely miR-579, no PCa related functions have been reported. MiR-579-3p is only known as a master regulator of melanoma progression and drug resistance [48].

Among the under expressed DE microRNAs mir-496, miR-137, miR-1, and miR-370 had the highest combined P-values across all studies.

MiR-496 is also a previously poorly characterized miRNA, which has no functions in PCa. Methylated DNA binding domain protein 2 (MBD2) is known as the only TF of miR-496, which coordinately silences gene expression through activation of the miR-496 promoter in breast cancer cell line [49].

Methylated mir-137 host gene is promising diagnostic and/or prognostic biomarker of PCa. The epigenetic silencing of miR137 is an important event in promoting androgen signaling during prostate carcinogenesis and progression. MiR-137 suppresses cell growth in several cancers such as ovarian, colorectal, and gastric [50].

MiR-1 is known as a biomarker of recurrence PCa, which is in agreement with the findings in present meta-analysis study. MiR-1 functions as a tumor suppressor which suppresses cancer cell proliferation, metastasis, angiogenesis, invasion, cell cycle arrest, WNT signaling and promotes apoptosis by ectopic expression. This miRNA is a potential prognostic biomarker of hepatocellular carcinoma (HCC) and colorectal cancer. The expression of miR-1 alters in several cancers such as lung, gastrointestinal, prostate, bladder, head and neck, and renal cancer [51].

MiR-370 plays an important role in the proliferation of human PCa cells by directly suppressing the tumor suppressor FOXO1 [39].

PPI Hub Proteins analysis of the TF and target genes of DE MicroRNAs was conducted for prioritization of the most important hub genes using the EnrichR web tool. CTNNB1 was the most important hub genes among TF and target genes of DE microRNAs across six microarray studies.

CTNNB1 (Catenin Beta 1) functions as a Key downstream component of the canonical WNT signaling pathway. WNTs and their downstream effectors have crucial roles in the regulation of various processes that are important for cancer progression, including tumor growth, tumor initiation, differentiation, cell senescence, cell death, differentiation and metastasis [52]. Nuclear accumulation and abnormal stabilization of CTNNB1 as a consequence of missense mutations occurs at a high frequency in a variety of epithelial cancers such as colorectal cancer, medulloblastoma, ovarian cancer, and pilomatrixoma. Upregulation of CTNNB1 is also associated with PCa [53].

To elucidate the role of DE microRNAs obtained from the meta-analysis, we performed pathway analysis and gene set enrichment analysis for TF and target genes of DE miRNAs using the EnrichR web tool. The most enriched pathway and Gene Ontology (GO) term among the TF and target genes of DE miRNAs were “MicroRNAs in cancer (hsa05206)”, “Pathways in cancer (hsa05200)”, Signal Transduction (R-HSA-162582)”, “regulation of epithelial cell proliferation (GO: 0050678)” and “tissue morphogenesis (GO: GO:0048729)”.

Common pathway analysis revealed that TCF3, MYC, MAX, CYP26A1 and SREBF1 were the most significant proteins associated with DE miRNA genes. Of note, these proteins were not identified as TF and target genes of DE microRNAs.

Previous studies have reported that the diminished activity of TCF3 plays a role in lymphoid malignancies, and up-regulation of it is involved in the development and progression of colorectal cancer. TCF3 is regulated by androgens and acts as a tumor promoter in PCa [54].

Overexpression, Mutations, translocation and rearrangement of MYC is related to several cancers such as breast, PCa, gastrointestinal, melanoma, and small cell lung cancer [55].

MAX is known as a tumor suppressor in renal oncocytomas and small cell lung cancer. The mutation of it has been identified in gastrointestinal stromal tumors [56]. High expression of CYP26A1 is associated with several cancers such as breast, head and neck, colorectal and ovarian. CYP26A1 is a methylation marker of PCs associated with ERG-positive cancers [57].

Sterol regulatory element-binding protein1 (SREBP1) is a key regulatory factor that controls lipid homeostasis. SREBP1 is a critical link between oncogenic signaling and tumor metabolism. The overexpression of SREBF1 is related to a variety of cancers such as PCa, breast, head and neck, colorectal, endometrial, glioblastoma, pancreatic, and ovarian [58].

To understand the association of the DE microRNAs list with the most significant target genes and transcription factors, we conducted a regulatory gene network analysis using the MIROB web tool. CDKN1A and LASP1 were amongst the most significant target genes associated with the DE microRNAs.

Cyclin-dependent kinase inhibitor 1 (CDKN1A) also is known as P21 is involved in p53/TP53 mediated inhibition of cellular proliferation in response to DNA damage and its overexpression results in cell cycle arrest and autophagy cell death. The expression of this gene is tightly controlled by the tumor suppressor protein p53 in a human brain tumor cell line [59]. The CDKN1A genotypes CT and TT are associated with an increased risk of advanced prostate carcinoma compared with the CC genotype [60]. Elevated p21 levels are associated with higher Gleason score, and increased PCa recurrence [61].

LIM and SH3 protein 1 (LASP1), a promoter of cell proliferation and migration, play a significant role in cancer development and progression. LASP-1 is involved in numerous biological and pathological processes. It plays an important role in the regulation of dynamic actin-based and cytoskeletal activities. LASP-1 is highly expressed in the central nervous system and contributes to the formation and progression of prostate cancer through a NF-KB pathway [62].

RELA, SNAI2, and TP53 were among the most significant transcription factors associated with the DE microRNAs.

RELA also known as NF-kappa-B is a ubiquitous transcription factor involved in many biological processes such as immunity, inflammation, cell growth, differentiation, tumorigenesis and apoptosis. Zinc finger protein (SNAI2) is known as a transcriptional repressor that modulates both activator-dependent and basal transcription. SNAI2 regulates cell proliferation and invasiveness of metastatic PCa cell lines. Cellular tumor antigen p53 (TP53) acts as a tumor suppressor in many tumor types; induces growth arrest or apoptosis depending on the physiological circumstances and cell type.

Moreover, network analysis showed that ten of the 37 DE miRNAs (miR-125A, miR-133B, miR-137, miR-221, miR-28, miR-340, miR-370, miR-449A, miR-874, and miR-98) have an established prognostic significance in other cancers such as colorectal, gastric, and breast. This network also indicated that eight of 37 DE miRNAs (miR-133A, miR-133B, miR-137, miR-199A1, miR-340, miR-361, miR-498, and miR-661) can be actively involved in tumor growth.

In this study, we also built new miRNA diagnostic classifiers in each GEO datasets based on best subset of DE miRNAs in the meta-analysis. These classifiers predicted BCR after RP with very high accuracy. The highest diagnostic accuracy (97%) was given for GSE55323 with 11-miRNAs. The performance of our 11-miRNA diagnostic classifier (97%) exceeded that of a 2-miRNA classifier (miR-1+miR-133B; AUC: 71%) developed earlier by Karatas *et al*. [10]. One miRNA (miR-1) is shared between these classifiers, further supporting the validity of our findings.

Briefly, we used “MetaDE” package to perform a meta-analysis, which provides options for gene matching across studies, gene filtering before meta-analysis and functions for conducting several major meta-analysis methods such as Fisher and AW for differential expression analysis. Then performed the GO enrichment analysis, pathway analysis, network analysis, and ROC analysis.

In conclusion, this is the first report that provides biological insights on common microRNA expression signatures for recurrent PCa after RP. The candidate miRNAs are worthy to be validated in the wet lab.

## Supporting information

S1 Fig The PRISMA flow diagram. (TIF)
